# Plant phenolics are detoxified by prophenoloxidase in the insect gut

**DOI:** 10.1038/srep16823

**Published:** 2015-11-23

**Authors:** Kai Wu, Jie Zhang, Qiaoli Zhang, Shoulin Zhu, Qimiao Shao, Kevin D. Clark, Yining Liu, Erjun Ling

**Affiliations:** 1 Key Laboratory of Insect Developmental and Evolutionary Biology, Institute of Plant Physiology and Ecology, Shanghai Institutes for Biological Sciences, Chinese Academy of Sciences, Shanghai 200032, China; 2 Department of Food Science and Technology, University of Georgia, Athens, GA 30602, USA; 3 Central Lab of Key Equipments, Institute of Plant Physiology and Ecology, Shanghai Institutes for Biological Sciences, Chinese Academy of Sciences, Shanghai 200032, China

## Abstract

Plant phenolics are a group of important secondary metabolites that are toxic to many animals and insects if ingested at high concentrations. Because most insects consume plant phenolics daily, they have likely evolved the capacity to detoxify these compounds. Here, we used *Drosophila melanogaster*, *Bombyx mori* and *Helicoverpa armigera* as models to study the metabolism of plant phenolics by prophenoloxidases. We found that insect foreguts release prophenoloxidases into the lumen, and that the survival of prophenoloxidase-deletion mutants was impaired when fed several plant phenolics and tea extracts. Using l-DOPA as a model substrate, biochemical assays in large *Lepidopteran* insects demonstrated that low levels of l-DOPA are rapidly metabolized into intermediates by phenoloxidases. Feeding with excess l-DOPA showed that the metabolic intermediate 5,6-dihydroxyindole reached the hindgut either by passing directly through the midgut, or by transport through the hemolymph. In the hindgut, 5,6-dihydroxyindole was further oxidized by prophenoloxidases. Intermediates exerted no toxicity in the hemocoel or midgut. These results show that plant phenolics are not toxic to insects unless prophenoloxidase genes are lost or the levels of phenolics exceed the catalytic activity of the gut prophenoloxidases.

Plants produce many secondary metabolites, such as alkaloids, glucosinolates, terpenoids, and phenolics, which can aid in defending against attacks by insect herbivores and pathogens^1^,^2^,^3^,^4^,^5^. Artificial feeding assays have indicated that most phenolics are toxic to numerous insects^1^,^2^,^3^,^4^,^5^,^6^,^7^,^8^. For example, feeding of high doses of l-DOPA to insects induces abnormal growth and development^5^,^9^,^10^,^11^. Gallic acid reduces the conversion efficiency of assimilated food in *Helicoverpa armigera* larvae, which also show continuous defecation and muscular lesions in the hindgut^7^. After intensive feeding, tannic acid can bind to insect gut tissue and cause damage^7^. Therefore, plant phenolics are considered to play a major role in plant defenses against insect predation^2^. However, most insects have to consume plant phenolics every day, and this raises the question as to whether they have evolved resistance to plant phenolics.

Insect prophenoloxidase (PPO), an important innate immunity protein^12^,^13^,^14^,^15^, belongs to a family of type 3 copper-containing proteins expressed in almost all organisms^13^. PPOs exist as zymogens that can be activated physiologically by specific proteases, or artificially by ethanol^14^. Once activated, hemolymph phenoloxidase (PO) can oxidize phenolics to induce melanization around invading bacteria and parasites^12^,^13^. In a previous study, we found that the foreguts of various insect species, including *Bombyx mori* and *H. armigera*, were stained black by substrates containing ethanol^16^. As ethanol specifically activates insect PPO but not other enzymes^14^,^17^, these observations suggested the presence of PPO in the insect foregut. Due to its capacity for metabolizing phenolics^18^, we hypothesized that foregut PPO may be involved in the detoxification of plant phenolics.

Here we used *Drosophila*, *B. mori*, and *H. armigera* as models to determine the presence of PPO in the insect foregut, and how it functions to detoxify plant phenolics. We found that PPO is released into the foregut lumen, binds to food fragments, and is then activated by an unknown serine protease(s). Active PO then oxidizes plant phenolics into intermediates without inducing melanization in the foregut or fore-midgut unless gut motility is impaired. These intermediates are transferred to the hindgut where they are continuously oxidized by hindgut PO into melanized materials. Plant phenolics are not toxic to insects if the level of foregut PPO is sufficient for their catalysis. However, intensive feeding may result in absorption of larger amounts of phenolics than normal, and thus cause damage if PPO is absent or PO activity is insufficient for the level of substrate. This work demonstrates that insect PPO is an important factor in detoxifying many plant phenolics.

## Results

### PPO is a key component for detoxification of phenolics

**Figure 1:**
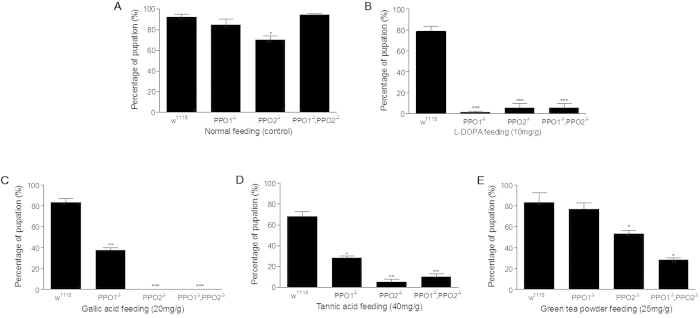
l-DOPA feeding affected the growth and development of PPO-mutant *Drosophila* larvae. *Drosophila* larvae (day 1 of first instar larval stage) of wild-type *w*^*1118*^ and the single (PPO1^Δ^ or PPO2^Δ^) and double (PPO1^Δ^/PPO2^Δ^) deletion mutants were fed a diet supplemented with nothing (**A**), l-DOPA (**B**), gallic acid (**C**), tannic acid (**D**) and green tea powder (**E**) as indicated. The feeding assays were repeated independently three times; in each replicate, 30 larvae were fed a diet supplemented with the indicated materials. An unpaired two-tailed *t*-test was performed to assess the significance of differences between groups unless otherwise stated. *P < 0.05; **P < 0.001; ***P < 0.0001.

Plant phenolics such as pure l-DOPA, gallic acid and tannic acid have been proven to be toxic to many insects^5^,^6^,^7^,^9^,^10^,^11^,^19^,^20^. Thus, they were selected to assess the importance of specific PPOs in detoxifying polyphenolic compounds in *Drosophila*. Groups consisting of identical numbers of *Drosophila* larvae (*w*^*1118*^, PPO1^Δ^, PPO2^Δ^, (PPO1^Δ^/PPO2^Δ^)) at the same age (first day of the first instar larval stage) were selected and fed a fresh diet supplemented either with or without l-DOPA, gallic acid, tannic acid and green tea powder separately. During the wandering stage, both wild-type and mutant larvae fed normal diet attained approximately the same sizes, and most reached the pupa stage. However, the pupation rate of the PPO2^Δ^ mutants was significantly lower than those of the other groups (Fig. 1A), indicating that PPO2 is important for development despite its lower activity than PPO1^21^. If l-DOPA was fed, few of the mutant larvae (PPO1^Δ^, PPO2^Δ^, (PPO1^Δ^/PPO2^Δ^)) reached the pupa stage because they died during the feeding assay (Fig. 1B). When either gallic acid (Fig. 1C) or tannic acid (Fig. 1D) was fed to *Drosophila* larvae, those mutants with single or double PPO genes deleted had significantly lower ratios of pupation. Very interestingly, when green tea powders were added to the diet, the pupation ratios of PPO2^Δ^, (PPO1^Δ^/PPO2^Δ^) were also significantly lower than the wild type (Fig. 1E). The PPO1^Δ^ mutant also had lower level of pupation than the wild type, but it was not significant.

**Figure 2:**
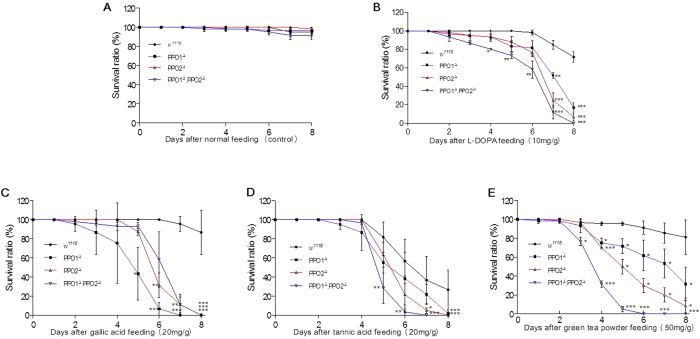
PPO is important for detoxification of phenolics by adult *Drosophila*. New adults (day 1 after eclosion) of *Drosophila* (*w*^*1118*^ and the PPO deletion mutants, as indicated) were fed a diet supplemented with nothing (**A**), l-DOPA (**B**), gallic acid (**C**), tannic acid (**D**) and green tea powder (**E**) as indicated. Living adults were counted daily, from which survival ratios were calculated. Feeding assays were repeated independently three times; in each replicate, 20 adults were fed a diet supplemented with the indicated materials. *P < 0.05; **P < 0.001; ***P < 0.0001.

After eclosion, identical numbers of new adults of the wild type and the three mutants were separately fed a diet supplemented either with or without phenolics (l-DOPA, gallic acid, tannic acid and green tea powder). There were no significant differences between the wild-type and the three mutants when fed the diet without supplement (Fig. 2A). However, when the diet contained excess l-DOPA, the number of surviving PPO1^Δ^/PPO2^Δ^ double mutants decreased significantly after the 4th day (Fig. 2B). Almost all adult PPO1^Δ^/PPO2^Δ^ double mutants were dead on the 8th day. The numbers of adults of the single PPO1 (PPO1^Δ^) and PPO2 (PPO2^Δ^) mutants also decreased during the period of the assay, and were significantly lower than that of the wild type by the 7th day.

Wild-type *Drosophila* adults fed gallic acid exhibited slightly decreased survival rates (Fig. 2C). However, when both PPO genes were deleted, over half of adults were dead on the 5th day and almost all were dead on the 7th day (Fig. 2C). Single PPO gene deletion mutants exhibited a decreasing survival ratio after the 4th day, and almost all were dead on day 8.

Tannic acid fed to wild-type *Drosophila* adults was highly toxic as shown by greatly decreased survival ratios on the 5th day (Fig. 2D). However, on each day, the survival ratios of adults with only a single PPO gene deletion were still lower than that of wild type. The survival ratios of PPO1^Δ^/PPO2^Δ^ double mutants were significantly lower than that of either the wild type or the single deletion mutants, with most being dead on the 8th day.

When green tea powder was mixed with the diet and fed to the *Drosophila* adults, the survival ratios of all mutants were significantly lower than that of wild type (Fig. 2E). Most adults containing the double PPO1^Δ^/PPO2^Δ^ deletions were dead after the 5th day.

These data indicated that PPOs are important for detoxifying l-DOPA, gallic acid and tannic acid in foods including phenolics in green tea. Intensive feeding of those phenolics was also toxic to wild type *Drosophila* (Figs 1 and 2), which is similar to other insects^5^,^6^,^7^,^9^,^10^,^11^. Due to the role of PPOs in detoxifying phenolics, we next investigated the metabolism of l-DOPA within the foregut.

### The insect foregut produces PPO that is secreted into the lumen

**Figure 3:**
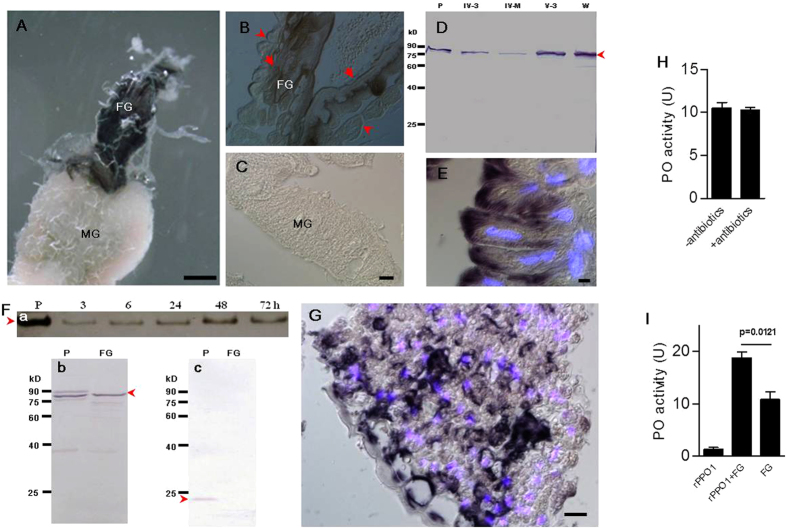
The silkworm foregut secretes PPO. A mixture of ethanol and l-DOPA was used to stain gut tissue (**A**), and the stained tissues were fixed and sectioned (**B**). Epidermal (arrow) but not muscle (arrowhead) cells were stained black (**B**). Cells in the fore-midgut (see Fig. 10 for the position) were not stained (**C**). (**D**) PPOs (arrowheads) were detected by Western blot in the larval foreguts. The location of PPO in foregut epidermal cells (**E**). PPO was detected by immunostaining using an antibody against *Bombyx mori* PPO^41^. DAPI was used to counterstain cell nuclei (blue). (**F**) PPO released from foregut epidermal cells. (**a**) Foreguts (V-3) were cultured for the indicated times. Tissue culture medium was separated on a native gel to show PPO position (arrowhead). (**b**) PPO (arrowhead) was detected in the foregut (FG) contents by Western blot. (**c**) Immune-inducible lysozyme (arrowhead) was detected by Western blot in plasma but not in the foregut contents of the same larvae. (**G**) PPO and/or PO bound to ingested mulberry leaf fragments in the crop. (**H**) PO activities of fresh foregut contents (1 μl) taken from larvae fed with antibiotics (+) or not (−) for 2 days. Bacteria were cleared from the gut if antibiotics were fed. (**I**) PO activities were enhanced when rPPO1 was incubated with fresh foregut contents (1 μl) for 30 sec. Columns represent the means of three independent measurements ± S.E. P, plasma. FG, foregut content. Bar: (**A**) 1 mm; (**B**, **C**,**E**,**F**) 10 μm.

The insect digestive system is composed of three parts: the foregut, midgut, and hindgut. The silkworm foregut has a long, tube-like esophagus and crop. When the foregut and midgut were stained by l-DOPA dissolved in ethanol, the foregut—but not the midgut—became melanized (Fig. 3A). The above tissues were then fixed and sectioned. Epidermal cells—but not muscle cells—in the foregut stained positive (Fig. 3B), while the neighboring midgut (fore-midgut) showed no staining (Fig. 3C). Foregut tissues from larvae at different developmental stages showed bands at the same position as plasma PPO (Fig. 3D). Large amounts of PPO were detected in the epidermal cells of the esophagus and crop (Fig. 3E). PPO1 mRNA was detected in the foregut, but that of PPO2 mRNA was weak (Fig. S1). No PPO mRNAs were detected in the midgut, which confirms previous results^16^. The silkworm larval foregut (V-3) was dissected for tissue culture, and PPO was detected in the culture medium (Fig. 3F-a). Foregut contents also contain PPO proteins (Fig. 3F-b). To ensure that the observed PPO was not due to hemolymph contamination, we tested for the presence of the plasma protein Lysozyme in foreguts. Lysozyme was readily detected in immune-challenged plasma but not in the foregut contents of the same larvae (Fig. 3F-c). Next, mulberry leaf fragments were extracted for immunostaining; foregut PPO/PO was found to bind to mulberry leaf fragments (Fig. 3G). A PPO/PO signal was also detected on food fragments in the fore-midgut. No silkworm PPO/PO signals were detected on fresh mulberry leaves.

The foregut contents had obvious PO activity (Fig. 3H), but whether POs are present to metabolize phenolics or as an immune response to gut bacteria is unclear. When antibiotics were fed to the silkworm larvae to clear all bacteria, there was no difference in PO activity in the foregut contents (Fig. 3H). PPO was not activated by microbes in the foregut. When *Drosophila* recombinant PPO1 (rPPO1) was incubated with the foregut contents for 30 sec, PO activity was significantly enhanced (Fig. 3I). Obviously, there is unknown mechanism to activate PPOs in the foregut.

In *Manduca sexta*, the proPO activating proteinase (PAP) cleaves PPO for activation^22^. The substrate acetyl-Ile-Glu-Ala-Arg-p-nitroanilide (IEAR) was used to detect the proPO activating proteinase (PAP) activity^22^. The foregut content had IEARase activity (Fig S2A). However, commercial tryspin and α-chymotrypsin also degraded IEAR. The foregut contents contained chymotrypsin-like enzyme activity, as shown using the specific α-chymotrypsin substrate benzoyl-L-tyrosine ethyl ester (BTEE)^10^,^11^ (Fig. S2B). α-chymotrypsin has been shown previously to activate rPPO1 *in vitro*^23^. We conclude that there may be unknown chymotrypsin-like protease(s) in the foregut to activate PPO.

**Figure 4:**
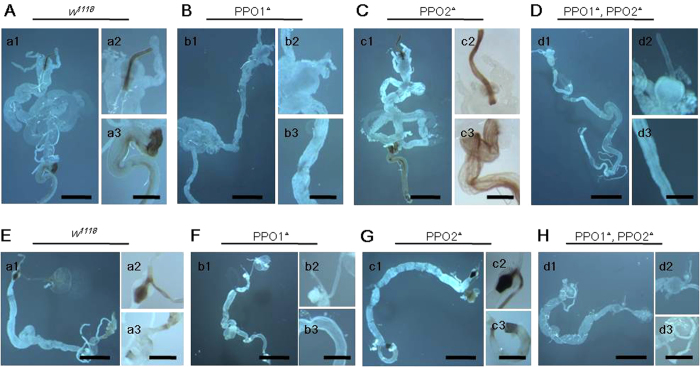
Detection of PPOs in the foreguts of *Drosophila* larvae (**A**–**D**) and adults (**E**–**H**). *w*^*1118*^ and PPO deletion mutants as indicated were dissected and the gut stained as in Fig. 1A. (**a1**,**b1**,**c1**,**d1**) Images of the whole gut after staining. (**a2**,**b2**,**c2**,**d2**) Enlarged images of parts of the foregut. (**a3**,**b3**,**c3**,**d3**) Enlarged images of parts of the hindgut. Bar: (**a1**,**b1**,**c1**,**d1**) 1 mm. (**a2**,**a3**,**b2**,**b3**, **c2**,**c3**,**d2**,**d3**) 0.25 mm.

The guts of *Drosophila* larvae (Fig. 4A–D) and adults (Fig. 4E–H) were also stained as described above (Fig. 3A). *Drosophila* foreguts (especially the esophagus) showed staining, indicating the presence of PPO in the larval (Fig. 4A-a1,A-a2) and adult (Fig. 4E-a1,E-a2) foreguts. The larval and adult hindguts also stained positively (Fig. 4A-a3,E-a3). *Drosophila* mutants in which two PPO genes (PPO1 and PPO2) had been deleted were reported recently^24^. In the PPO1 deletion mutant (PPO1^Δ^), the larval and adult foreguts and hindguts showed weak staining (Fig. 4B-b1–b3). However, the PPO2-deletion mutants (PPO2^Δ^) showed staining in the larval and adult foreguts and hindguts (Fig. 4C-c1–c3). In PPO1 and PPO2 double mutants (PPO1^Δ^/PPO2^Δ^), no staining was detected in the larval and adult foreguts and hindguts (Fig. 4D-d1–d3). An immunoblot showed that both PPO1 and PPO2 were present in the foregut of *Drosophila* larvae (Fig. S3). These results indicated that the insect foregut contained PPO.

**Figure 5:**
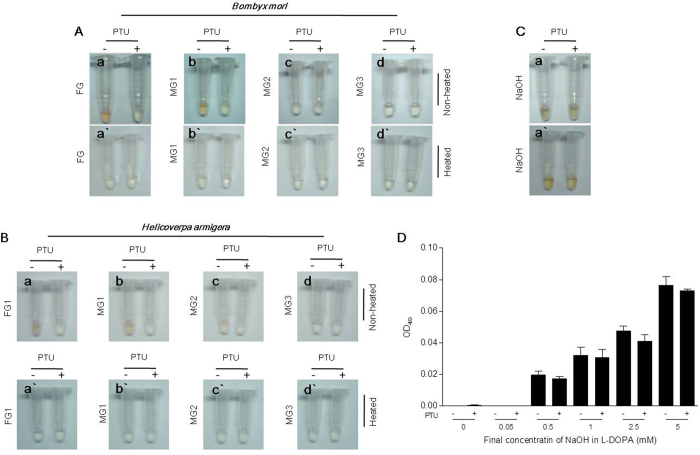
PPO in the foregut lumen. In *Bombyx mori* (**A**) and *Helicoverpa armigera* (**B**), supernatants prepared from the contents of the foregut (FG) and fore-midgut (MG1) , but not the middle-midgut (MG2) or hind-midgut (MG3), showed PO activity, which was inhibited by PTU. Supernatants (1 μl) from different parts of the gut, or an alkaline solution ((**C)** 500 mM NaOH, 1 μl), were mixed with 100 μl of l-DOPA (2 mM) for 10 min. (**a′**–**d′**) Before mixing with substrates, supernatants from the corresponding gut contents or a NaOH solution were heated at 100 °C for 5 min to deactivate enzymes. (**D**) High concentrations of NaOH oxidized l-DOPA. NaOH at the indicated concentrations was mixed with l-DOPA and incubated for 5 min to read the absorbance (OD_490_). Low concentrations of NaOH (<0.05 mM) did not oxidize l-DOPA. The gut structure is presented in Fig. 10. FG, foregut; MG1, fore-midgut; MG2, middle-midgut; MG3, hindgut-midgut.

PPOs in the foregut contents (pH 7–8) of *B. mori* and *H. armigera* oxidized substrate, thus exhibiting PO activity (Fig. 5A-a,B-a), which was inhibited by PTU. However, gut contents from the fore-midgut, middle-midgut, and hind-midgut may be alkaline, which could result in the non-enzymatic oxidation of phenolics. Sodium hydroxide (NaOH) at a concentration higher than 0.5 mM (>pH 10) caused oxidization of l-DOPA that was not inhibited by PTU (Fig. 5C-a,D). When NaOH was 0.05 mM (pH 9), it did not oxidize l-DOPA (Fig. 5D). Fore-midgut—but not middle-midgut or hind-midgut—contents oxidized substrate similar to the foregut contents (Fig. 5A-b–A-d,B-b–B-d). Due to the lack of PPO in the fore-midgut (Fig. 3C), PPO in the fore-midgut contents is likely derived from the foregut. Heating had no effect on substrate oxidation by NaOH (Fig. 5C-a′ and Fig. S4), but eliminated gut-content-mediated substrate oxidation (Fig. 5A-b′–A-d′). The contents of the middle-midgut and hind-midgut may not oxidize substrates efficiently due to low concentration of hydroxyl ion or a lack of oxygen. No obvious laccase or peroxidase activities were detected in the foregut contents (Fig. S5). These observations indicated that PPO is released from the foregut into the lumen.

### Plant phenolics are substrates for insect PO *in vitro*

**Figure 6:**
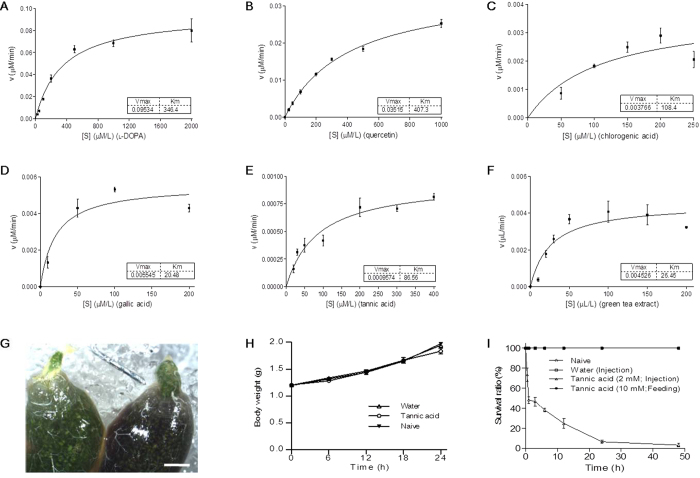
Plant phenolics are substrates for insect PPOs. (**A**–**F**) Enzyme kinetics were measured using different phenolics as substrates. Each substrate at the indicated concentrations was mixed with ethanol-activated rPO1 (0.5 μg). Maximum absorbances were read at wavelengths specific to the products of each substrate (see Material and Methods for detail). The calculated Vmax and Km values are indicated in each figure. (**G**) Food in the foregut and fore-midgut did not become melanized unless the larvae were placed on ice for 2 h to stop gut motility. No melanization was observed in a freshly dissected gut (0 h). (**H**) Tannic acid was non-toxic following feeding to silkworm larvae. Larvae (IV-1) were fed mulberry leaves supplemented with 10 mM tannic acid or water (control) and weighed every 6 h. Each group consisted of 10 individuals. (I) Tannic acid (2 mM; 20 μl) or sterile water (20 μl) was injected into the hemocoel of silkworm larvae (IV-1), or larvae were fed mulberry leaves coated with 10 mM tannic acid, as described above. The survival ratios were calculated at the indicated times. Columns represent the means of three independent measurements ± S.E. Each group consisted of 10 (**H**) or 20 (**I**) individuals. Bar, 2.5 mm.

As PPO was shown to be present in the insect foregut, we next determined whether it accepts plant phenolics as substrates. l-DOPA, gallic acid, tannic acid, chlorogenic acid, and quercetin are important plant phenolics^1^,^2^,^3^,^4^,^5^. When used as substrates separately, each was oxidized by activated rPO1 (Fig. 6A–E). The phenolics in tea extracts were also oxidized by activated rPO1 (Fig. 6F). Therefore, plant phenolics are substrates for insect PPO. Mulberry leaves also produce many phenolics that may be oxidized by PPOs^1^,^2^,^3^,^4^,^5^. To test this, silkworm larvae were fed mulberry leaves, then placed on ice for 2 h. Both the foregut and fore-midgut contents of the larvae, regions positive for PO activity (Fig. 5A-a,A-b), became melanized (Fig. 6G), suggesting that plant phenolics may be metabolized into intermediates but not oxidized to terminal melanins unless gut motility is impaired through placing on ice. The melanization shown in Fig. 6G is not due to plant-derived polyphenol oxidases since similar melanization was observed when cooked diet was fed to larvae before placement on ice. Tannic acid is another plant-derived phenolic that is toxic to both insects and vertebrate herbivores^20^. Silkworm larvae fed mulberry leaves coated with tannic acid showed similar increases in body weight to those fed untreated mulberry leaves or leaves coated with water alone (Fig. 6H). However, most larvae died within 50 h following injection of tannic acid (2 mM, 20 μl) into the larval hemocoel (Fig. 6I). The larvae injected with water grew normally, similar to naïve larvae and those fed a high concentration of tannic acid. These results suggested that foregut POs play an essential role in metabolizing plant phenolics.

### l-DOPA is metabolized by PO in the foregut

The metabolism of l-DOPA by POs has been studied extensively in the context of insect immunity, and much is known about the intermediates produced. Because l-DOPA has been shown here and in other studies to be deleterious to insect viability^5^,^9^,^10^,^11^, we selected it to study the mechanism of phenolic detoxification within the insect gut. One of the intermediates derived from PO catalysis of l-DOPA *in vitro* is 5,6-dihydroxyindole (DHI), which we used as an indicator of metabolism. We first examined the production of intermediates *in vitro* with the intention of assessing the influence of PTU (a PO inhibitor). PPO was first activated by treatment with ethanol, or AMM1, or α-chymotrypsin, as described previously^21^,^23^. Ethanol induces significantly higher levels of PO activity than AMM1 and α-chymotrypsin. A number of the intermediates catalyzed by PO could be identified: 17 produced by ethanol-activated PO, 17 produced by AMM1-acivated PO, and 11 produced by α-chymotrypsin activated PO. When PTU was added, reduced levels of intermediates such as 5,6-dihydroxyindole (DHI) were produced (Fig. S6), indicating that the metabolism of l-DOPA by PO was not totally inhibited by PTU, although the amounts were low and melanins were not produced.

The three *Drosophila* PPO deletion mutants are good models for evaluating l-DOPA metabolism in the gut. However, due to their small sizes, it is difficult to collect gut contents without contamination. Therefore, large lepidopteran insects (*B. mori* and *H. armigera*) were used to assess metabolism of plant phenolics in the gut. For simplicity, an l-DOPA solution (10 μl) was fed directly to the larvae as indicated. After feeding with l-DOPA or water, the foregut was dissected 2 min later and the contents were removed immediately to assess levels of l-DOPA and its metabolites using mass spectrometry. l-DOPA (Fig. S7A-a) and DHI (Fig. S7B-a) solutions were used as standards. After feeding, l-DOPA was not detected in the foregut contents (Fig. S7A-b). However, low levels of DHI were detected (Fig. S7B-b). In contrast, no l-DOPA or DHI was detected in the water-fed or naïve larvae (Fig. S7A-c, S7A-d, S7B-c, and S7B-d). As indicated above, some unknown intermediates are likely produced in the foregut after l-DOPA feeding, but at levels below the limit of detection.

**Figure 7:**
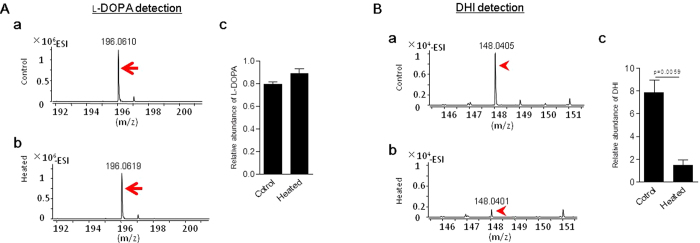
PO-mediated metabolism of l-DOPA by silkworm foregut juice. (**A**,**B**) Foregut contents were heated to deactivate enzymes. Non-heated foregut contents were used as the control. Identical volumes of heated and non-heated foregut contents were mixed with 200 μl of l-DOPA (2 mM) and allowed to react for 10 min. The l-DOPA (**A-a**,**A-b**) and DHI (**B-a**,**B-b**) spectra were detected separately. The relative l-DOPA (**A-c**) and DHI (**B-c**) levels in the reactions were compared separately. The arrows indicate l-DOPA and the arrowheads indicate DHI spectra. Each column represents the mean of three independent measurements ± S.E.M.

We next assessed the contribution of foregut contents to l-DOPA metabolism. Foregut contents were removed without hemolymph contamination (Fig. 3F-c), then one aliquot was heated to deactivate enzymes. The heated and non-heated (control) foregut contents were mixed with excess l-DOPA separately, incubated for 10 minutes, and the amount of l-DOPA was determined (Fig. 7A-a,A-b). The level of l-DOPA was lower in the non-heated mixture (Fig. 7A-c). In addition, a significantly higher level of DHI was produced by the non-heated mixture (Fig. 7B-a,B-b,B-c). These data indicated that the foregut content PO, but not the foregut content supernatant with POs denatured through heating beforehand, catalyzes the metabolism of phenolics both *in vivo* and *in vitro*. Metabolism therefore does not occur through a non-enzymatic process.

Obviously, l-DOPA can be metabolized by foregut PO to produce intermediates, among which DHI was detected (Fig. S7B-b). We next sought to address whether these intermediates disappear in the midgut, and whether they are cytotoxic *in vivo*.

### Intermediates pass through the midgut and are partially absorbed into the hemolymph

In the above assay, the silkworm larvae were unable to consume more than one drop of solution. The small amount of l-DOPA present in this feeding solution was not sufficient to allow tracking of l-DOPA and/or other intermediates in the silkworm midgut. The omnivorous lepidopteran insect, *H. armigera*, which is capable of consuming larger volumes, was therefore selected for tracking l-DOPA and its intermediates in the body. As a control, the midgut contents of *H. armigera* larvae were removed and heated to inactivate enzymes. l-DOPA solution was added to the heated contents, but no DHI was produced after incubation, indicating that the heat-inactivated *H. armigera* midgut contents did not significantly convert l-DOPA into intermediates (Fig. S8). To show that phenolic metabolism does occur in *H. armigera,* larvae were initially fed an artificial diet and then transferred onto green leaves. Within 3 h, the midgut contents changed from brown to green due to the altered diet. A 3-h feeding period was sufficient to detect the intermediates produced in the content as a result of metabolism of phenolics.

**Figure 8:**
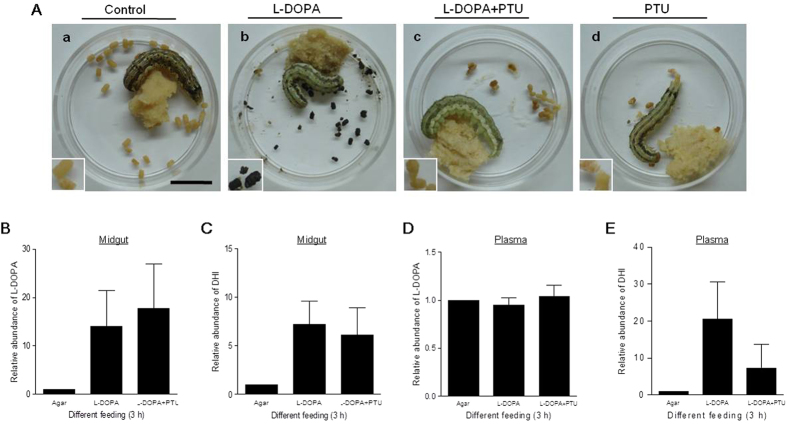
Detection of intermediates metabolized from l-DOPA in the gut. (**A**) l-DOPA feeding was the main factor inducing melanization of feces, an effect that was inhibited by PTU. *H. armigera* larvae (V-1) were fed diets containing the indicated supplements. Insets show enlarged photographs of feces following each treatment. (**B**–**E**) Detection of l-DOPA and DHI in the midgut contents (**B**,**C**) and plasma (**D**,**E**), respectively. *H. armigera* larvae (V-1) were fed agar containing the indicated supplements for 3 h. Larval midgut contents and hemolymph were then sampled for mass spectrometry. Each column represents the mean of three independent measurements ± S.E.M. Bar: 1 cm.

Upon supplementation of the artificial diet with excess l-DOPA, *H. armigera* larvae excreted black but not brown feces (Fig. 8A-a,A-b); this phenomenon was inhibited by the PO inhibitor PTU (Fig. 8A-c). PTU alone had no effect on the feces color change (Fig. 8A-d). The artificial diet used in this assay contains many small molecules that could interfere with detection of intermediates. To replace the artificial diet, larvae were fed solidified agar alone or agar supplemented with l-DOPA+/− PTU. When *H. armigera* larvae were fed agar alone, little l-DOPA or DHI was detected in the midgut contents or plasma (Fig. 8B–E). Supplementation of the agar with excess l-DOPA or l-DOPA plus PTU resulted in detection of both l-DOPA (Fig. 8B) and DHI (Fig. 8C) in the midgut contents. Their levels in the midgut contents were similar to, but higher than, those in the group fed agar alone (Fig. 8B,C). The l-DOPA signal in plasma was similar among three feeding treatments (Fig. 8D), indicating that l-DOPA did not accumulate in the hemolymph. DHI was also detected in the plasma (Fig. 8E). In the group fed both l-DOPA and PTU, the DHI level in plasma was lower than that in the group fed l-DOPA alone (Fig. 8E), which is similar to that in the midgut (Fig. 8C). Although PTU inhibited the melanization of feces (Fig. 8A-c), it did not inhibit the production of intermediates such as DHI *in vivo* (Fig. 8C) as well as *in vitro* (Fig. S6). When l-DOPA plus PTU was fed, there was DHI detected in the freshly excreted feces. After feeding excess l-DOPA, *H. armigera* pupae were very small. Foregut-derived PO is likely a major factor in metabolizing l-DOPA to DHI, but the alkaline environment and other unknown factors may also promote production of DHI from l-DOPA. These data indicated that metabolic intermediates such as DHI were present in the midgut contents and in the feces of larvae fed excess l-DOPA. In addition, DHI was transferred into the hemolymph. DHI is cytotoxic *in vitro*^25^. Can DHI induce cytotoxicity *in vivo*?

**Figure 9:**
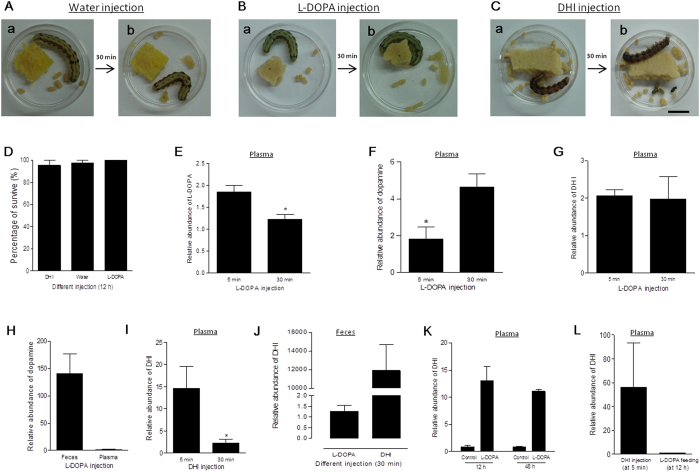
Hemolymph DHI was transferred into the hindgut for excretion without causing cytotoxicity *in vivo*. Identical volumes of sterile water (**A**), l-DOPA (**B**), and DHI (**C**) were injected into *H. armigera* larvae (V-1) respectively. The excreted feces were compared before and 30 min after injection. (**D**) The survival rates of *H. armigera* larvae did not differ among the treatments. (**E**–**H**) l-DOPA in hemolymph introduced through injection was metabolized into dopamine but not DHI through DDC. *H. armigera* larvae were injected with l-DOPA. At 5 and 30 min post injection, plasma was obtained to determine plasma l-DOPA (**E**), dopamine (**F**), and DHI (**G**) levels. (**H**) At 30 min, the dopamine levels in feces and plasma were also compared. (**I**–**K**) DHI is not cytoxicity *in vivo*. (**I**–**J**) DHI was excreted from hemolymph into feces. *H. armigera* was injected with DHI as shown in (**C**). At 5 and 30 min post injection, plasma was obtained to determine DHI (**I**). (**J**) The relative amounts of DHI in the feces from larvae that received l-DOPA and DHI injections were compared. (**K**) The DHI levels in larval plasma were determined after being fed a normal diet (control) or a diet supplemented with l-DOPA at 12 and 48 h, respectively. (**L**) Plasma DHI levels were compared between *H. armigera* larvae that underwent DHI injection at 5 min or l-DOPA feeding for 12 h. Each column represents the mean of three independent measurements ± S.E.M. *P < 0.05. Bar: 1 cm.

Intermediates derived from l-DOPA metabolism are not cytotoxic *in vivo*. Identical volumes of l-DOPA (2 mM), DHI (5 mM) and water were injected into the hemocoel of *H. armigera* larvae separately. In the water injection controls, the color of feces did not change (Fig. 9A). Nor was there an obvious change in feces color when l-DOPA was injected (Fig. 9B). However, following injection of DHI, the larvae excreted black feces within a limited period (Fig. 9C), which suggested that DHI can be transported into the hindgut and converted.

DHI was detected in the hemolymph of *Drosophila* after being infected by parasites^26^. When l-DOPA, DHI and water were injected separately, there were no significantly differences in survival ratios (Fig. 9D). Plasma l-DOPA and/or DHI were assayed at 5 and 30 min after injection of the corresponding molecules, as described above. Following l-DOPA injection, the plasma l-DOPA level decreased significantly from 5 to 30 min (Fig. 9E). Dopamine produced from injected l-DOPA via DOPA decarboxylase (DDC) catalysis^11^ was produced rapidly (within 5 min). The plasma dopamine level increased significantly within 30 min (Fig. 9F). DHI levels in plasma were stable (Fig. 9G). The relative abundance of dopamine in feces at 30 min was higher than that in plasma (Fig. 9H), which indicates that dopamine catalyzed from l-DOPA was transported into the feces. However, the actual amount of dopamine catalyzed from injected l-DOPA within this short period was probably too low to induce feces to melanize. Thus, l-DOPA was mainly metabolized into dopamine but not DHI in hemolymph. DHI detected in the midgut and plasma after feeding l-DOPA (Fig. 8) was produced in the foregut.

Following DHI injection, the plasma DHI level decreased significantly from 5 to 30 min (Fig. 9I). According to Fig. 9B,C, plasma DHI introduced through injection was excreted into the hindgut to induce feces into melanization. Significantly less DHI was detected in the feces if l-DOPA was injected (Fig. 9J). The plasma DHI levels at 12 and 48 h after feeding with l-DOPA were almost identical (Fig. 9K), indicating that DHI was continuously absorbed from the gut into the plasma and excreted into the hindgut at the same time. However, comparison of plasma DHI levels in the groups that underwent injection into the hemocoel at 5 min and l-DOPA feeding at 12 h indicated that the level of DHI absorbed (metabolized from l-DOPA feeding) was lower than that injected (Fig. 9L). Since injection of DHI did not significantly kill the insects (Fig. 9D), DHI is not cytotoxic *in vivo*.

**Figure 10:**
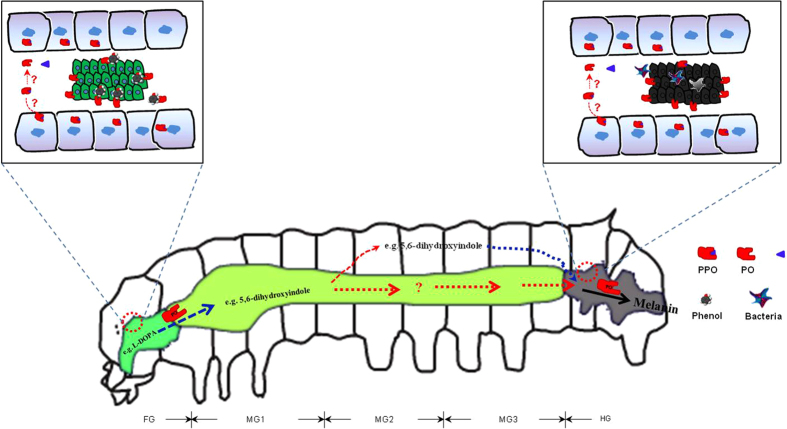
A schematic illustrating the detoxification of phenolics by insect PPOs. PPOs are released into the foregut lumen, where they are probably activated by chymotrypsin-like protease. Moderate levels of phenolics in food (e.g. l-DOPA) are metabolized by POs in the foregut and fore-midgut into various intermediates (e.g., DHI), which then pass through the midgut to reach the hindgut. Some intermediates are absorbed into the hemolymph. DHI is not cytotoxic as it cannot be spontaneously oxidized *in vivo*; hemolymph DHI is then transferred into the hindgut, probably via the Malpighian tubules. In the hindgut, intermediates such as DHI are oxidized by hindgut POs. FG, foregut; MG1, fore-midgut; MG2, middle-midgut; MG3, hindgut-midgut; HG, hindgut.

These data indicated that insect PPO is important for detoxifying food phenolics. Using l-DOPA as a PPO substrate, and DHI as a metabolic indicator, we found that phenolics (e.g. l-DOPA) can be metabolized by foregut PO to produce intermediates. Some intermediates (e.g. DHI) were transferred to the hindgut directly. The others were absorbed into hemolymph first and then transferred into the hindgut again, probably via Malpighian tubules, where they are oxidized by hindgut POs to induce feces melanization (Fig. 10).

## Discussion

Plant phenolics are important secondary metabolites^1^,^2^,^3^,^4^,^5^, most of which have beneficial effects on human health due to their antioxidant activity^4^. However, phenolics induce genotoxicity and thyroid toxicity in humans if ingested in large amounts^27^,^28^. Feeding assays have shown that phenolics such as gallic acid, salicylic acid, tannic acid, and l-DOPA have inhibitory effects on insect growth^5^,^6^,^7^,^9^,^10^,^11^. In the field, insects can adapt to the presence of plant phenolics, indicating that they have evolved specific mechanisms to detoxify or tolerate such compounds.

l-DOPA is found in many plants^5^, and its metabolism by POs has been studied in greater detail compared with that of other phenolics^18^,^29^. Therefore, use of food supplemented with l-DOPA is suitable for assaying the detoxification mechanism of foregut PO. In PPO1 and/or PPO2 *Drosophila* deletion mutants, the development and growth of larvae and adults fed a diet supplemented with l-DOPA were reduced markedly (Figs 1B and 2B). Gallic acid and tannic acid supplemented foods were also toxic to *Drosophila* adults with single or double PPO genes deleted (Fig. 2C,D). Therefore, insect foregut PPO is important for detoxifying phenolics.

*H. armigera* larvae continuously fed excess l-DOPA did not die, in contrast to the *Drosophila* PPO deletion mutants. However, their development was markedly affected. *H. armigera* pupae were smaller after being fed a diet supplemented with excess l-DOPA. Some insects, including *Drosophila*, express multicopper oxidases (MCOs) and a dual oxidase (Duox) that has a peroxidase homology domain^30^ in the gut that can oxidize l-DOPA and dopamine^31^,^32^. However, MCO1^31^ and Duox^30^ cannot rescue the toxicity induced by supplementation of the diet with l-DOPA in the PPO1^Δ^/PPO2^Δ^ mutant (Figs 1B and 2B). In *B. mori* and *H. armigera*, there may be peroxidases and/or laccases in the foregut contents. However, the activities of those enzymes were extremely low (Fig. S5). Thus, detoxification of plant phenolics by peroxidase and/or MCOs is not significant. In the insect midgut, there are many bacteria that confer resistance to nutritional depletion and other environmental stresses^33^. Midgut bacteria, which could also contribute to detoxifying phenolics, were separated and temporarily cultured with different substrates, but no obvious oxidization was observed. We also fed *Drosophila*, *B. mori* and *H. armigera* larvae with antibiotics to remove microbiota prior to dietary supplementation with different phenolics feeding, but again no obvious changes were observed. Therefore, the midgut microbiota did not detoxify phenolics. Although PTU inhibits both PO activity and melanization of feces in *H. armigera* (Fig. 8A), it cannot replace the effects of PPO deletion (PPO1^Δ^/PPO2^△^) since intermediates such as DHI were still produced, albeit at lower levels, *in vitro* and *in vivo* upon addition of PTU. Thus, PPO is a key factor for detoxification of plant phenolics by insects.

Upon feeding with excess l-DOPA, the *H. armigera* plasma l-DOPA level was not increased markedly compared with those fed the control diet (agar) alone (Fig. 8D). However, following injection of l-DOPA into the hemocoel, plasma l-DOPA levels decreased within 30 min (Fig. 9E). DDC is present in many insect tissues and likely contributes to l-DOPA metabolism in plasma. For example, l-DOPA levels were increased fivefold in a *Tribolium castaneum* mutant containing a DDC knockdown^34^, thus indicating the importance of DDC. In agreement with these results, we found that injected l-DOPA was catalyzed into dopamine by DDC in the hemocoel (Fig. 9F). Deletion of PPO1 and/or PPO2 in *Drosophila* resulted in absorption and transfer into the hemolymph of larger quantities of l-DOPA, where it was metabolized by DDC to produce dopamine and other compounds^18^. Elevated concentrations of the neurotransmitter dopamine impair neural activity^35^, which is probably responsible for the direct toxicity of l-DOPA when fed to insects. Silkworm larvae survive being fed a diet containing tannic acid, but die following a single injection into the hemocoel of even a small quantity (Fig. 6I). The intermediates of phenolics produced by PO exert no toxic effects in the midgut and hemocoel as they lose their capacity for spontaneous oxidization *in vivo*. We concluded that absorption of intact phenolics may result in toxicity in insects. It is unclear whether low levels of melanization occur when phenolics are metabolized by foregut POs *in vivo*. However, the catalase and superoxide dismutase present in the gut (Fig. S9) can act to reduce the levels of reactive oxygen species produced during melanization.

In this study, we were unable to assess all plant phenolics but utilized l-DOPA, gallic acid and tannic acid as a model to study detoxification by foregut PPO. The results presented here support the idea that insects use PPOs to detoxify food phenolics in the foregut to produce intermediates that will then be transferred into the hindgut and used for the inhibition of potential pathogen growth in feces^16^. These observations support the idea that insect PPOs have additional functions beyond immunity^14^. PPOs are likely one of the main factors involved in maintenance of the evolutionary stability of insect species. Any loss-of-function mutations in PPO genes will likely be lethal due to inability to metabolize plant phenolics. In the field, the levels of phenolics in food are probably appropriate for metabolism by foregut PPO, and so they are not absorbed and do not induce cytotoxic effects. However, when continuously ingested at high levels, the quantity of plant phenolics may exceed the metabolic capacity of foregut PPOs, resulting in cytotoxicity. To illustrate this, small mammals and insects will not consume Velvet bean seeds, which contain 4–7% l-DOPA, a level that is likely toxic^11^. Thus, prophenoloxidase is the main factor involved in detoxification of plant phenolics by insects, an important process in maintaining the balance of insect-plant interactions in the ecosystem.

## Methods

### Insect feeding and dissection

*B. mori* larvae (Nistari) were reared on mulberry leaves at 25 °C under a 12-h photoperiod. *Drosophila* (*w*^*1118*^, PPO1^△^, PPO2^△^, PPO1^△^/PPO2^△^) and *H. armigera* were fed on artificial diets: the standard diet for *Drosophila* contained agar, maize powder, sugar, yeast, nepagin, soyflour and propionic acid^36^; the standard diet for *H. armigera* contained wheat germ, yeast, methyl parahydrobenzoate, sorbic acid, ascorbic acid, linoleic acid, and agar^37^. To obtain microbe-free silkworm, larvae were fed mulberry leaves coated with antibiotics (Streptomycin), while *Drosophila* were fed diet supplemented with antibiotics. *Drosophila* wild-types and mutants with single or double PPO deletions were used for phenolic feeding experiments^24^. Silkworm larvae on day 1 and 3 of the fourth larval stage (IV-1, IV-3), the fourth molting stage (IV-M), day 3 of the fifth larval stage (V-3), and the wandering stage (W) were sampled for various experiments as needed. Larvae at the scheduled time were bled first and dissected in a sterilized 0.85% NaCl solution. The dissected tissues were washed in fresh 0.85% NaCl solution three times to remove hemocytes and plasma proteins and then used immediately.

### Gut content collection and PPO activation

To obtain gut contents, the gut was first washed in 0.85% NaCl three times. Then the gut was dried, cut open, and the contents from different parts transferred to separate pre-weighted tubes. Gut contents were suspended in either 10 mM Tris buffer (pH 7.4) (for enzyme activity assays) or a buffer containing 10 mM Tris (pH 7.4), 500 mM NaCl, 1 mM PMSF and 5 mM EDTA (for Western blotting) on ice. The tubes were vortexed several times, centrifuged at 10,000 × g at 4 °C for 5 min, and the supernatant was transferred to a new tube for subsequent use. The volume of the original gut contents was used for all calculations or comparisons unless otherwise mentioned. When the foregut contents were incubated at room temperature for 15 min, the foregut PPOs were totally degraded when assessed by immunoblot using a PPO-specific antibody. Recombinant *Drosophila melanogaster* PPO1 (rPPO1) was purified as described^21^. rPPO1 was activated by 30% ethanol or α-chymotrypsin or AMM1 as described^21^,^23^. PO activity assay and detection of active PO bands in the native-PAGE gel were performed as previously described^23^.

### Tannic acid feeding and injection into *B. mori*

Tannic acid (403040; Sigma) (10 mM, 100 μl) or water was applied to each piece of mulberry leave fragment (approximately 10 cm^2^) and allowed to air-dry. Twenty silkworm larvae (on IV-1) were continuously fed on the treated leaves for 2 days. As a control, tannic acid (2 mM, 20 μl) prepared in sterilized water was injected into staged larvae of equal ages. In each experimental repetition, 20 silkworm larvae received injections. Feeding and injections were repeated independently three times. After feeding and injections, the number of dead larvae was counted and percentages were calculated. Silkworm larvae fed with mulberry leave coated with tannic acid or water were weighed every 6 h for comparison.

### Gut staining

The dissected guts of the silkworm larvae (V-3) were incubated in 2 mM l-DOPA (ab120573; Abcam) solutions containing 30% ethanol. l-DOPA is a PPO substrate and ethanol is used to specifically activate PPO^13^. After staining, the guts were observed and imaged by microscopy. The melanized silkworm foregut was also fixed for tissue sectioning.

### Immune challenge and lysozyme detection

Silkworm larvae (V-3) were injected with 5 × 10^6^ formalin-killed *Escherichia coli* cells suspended in a sterilized 0.85% NaCl solution or in 0.85% NaCl alone. After 12 h larvae were bled to collect hemolymph. Cell-free plasma (supernatant) was prepared by centrifuging hemolymph at 5,000 × g for 5 min. Foregut contents were also sampled from the same larvae.

### Tissue culture and native gel analysis

Foreguts dissected from V-3 larvae were cultured in Grace’s medium containing 10% fetal bovine serum. The culture medium was sampled at different times for the appearance of PPOs by native gel electrophoresis. PPO activity was determined by adding l-DOPA to the gels as described previously^38^.

### Oxidization of plant phenolics

Some phenolics (l-DOPA, tannic acid, gallic acid, quercetin and chlorogenic acid) were selected as substrates for measuring enzyme kinetics. rPPO1 (0.5 μg) was activated using ethanol^21^. PO activity was measured by mixing each substrate at different concentrations with rPPO1. Tea leaves contain many phenolics^19^. To extract crude phenolics, Chinese green tea (1 g, Qingdao Fengli) was steeped in 20 ml boiled water until the temperature decreased to 25 °C. Tea extracts were centrifuged to remove debris at 5,000 × g for 3 min. The supernatant containing tea-derived phenolics was then used to measure PO enzyme kinetics. For each substrate or plant crude phenolics, the wavelength corresponding to the maximum absorbance of products was determined by scanning between 190 to 890 nm using a Libra S21 Visible Spectrophotometer. The maximum wavelengths for the products of each substrate were: l-DOPA, 490 nm; gallic acid, 332 nm; tannic acid, 362 nm; quercetin, 330 nm; chlorogenic acid, 400 nm; green tea, 362 nm. Absorbances were continuously read for 20 min on a Varioskan™ Flash Multimode Reader (Thermo Fisher Scientific Inc.). Analysis of kinetic data was performed by nonlinear regression to fit the data to the Michaelis–Menten equation: *V* = *V* max[*X*]/(*K*m + [*X*]) by using GraphRad Prism 5 (*V*, velocity; *V*max, maximal velocity; *K*m, Michaelis Constant; *X* , substrate concentrations). The kinetic parameters were determined from three independent experiments.

### Tissue fixation, sectioning and immuno-staining

Different parts of the silkworm gut were dissected as described above and fixed overnight at 4 °C in Bouin’s fluid^39^. Mulberry leave fragments were removed from different parts of the gut and fixed. Fresh mulberry leave fragments were also fixed and sectioned as controls. The above tissues were sectioned and paraffin was removed as described^40^.

To detect insect PPO in the larval foregut (V-3) or on ingested mulberry leave fragments from different parts of the gut, a polyclonal antibody against the silkworm PPO (a gift from Dr. T. Asano; 1:1,000) was used as the primary antibody^41^, and alkaline phosphatase conjugated goat anti-rabbit IgG (1:200) was used as the secondary antibody. Bands were visualized by the colorimetric reaction with bromo- chloroindolyl phosphate/nitro blue tetrazolium as the substrate. DAPI was used to counter-stain nuclei. All pictures were taken under a fluorescent microscope (Olympus BX51) under differential interference contrast using the appropriate filter.

### SDS-PAGE and Western blot analysis

Different parts of the silkworm or *Drosophila* larvae gut were dissected and homogenized and sonicated in 10 mM Tris-HCl, 1 mM PMSF (pH 7.4), and centrifuged at 10,000 × g at 4 °C for 5 min to collect supernatants. Protein concentrations were determined using the Bradford Protein Assay. Approximately 10 μg of protein was loaded per lane, then separated by 12% SDS-PAGE, transferred to nitrocellulose filter membrane and immunoblotted to visualize PPOs. Antibodies against either silkworm PPO (1:5,000)^41^, or lysozyme (a gift from Dr. K. Suzuki; 1:5,000)^42^ were used as primary antibodies, and an AP-conjugated goat anti-rabbit IgG (1:5,000) was used as the second antibody. Plasma (0.5 μl) from naïve larvae (V-3) was loaded as an indicator of PPO migration.

### Phenolics feeding

l-DOPA, gallic acid, tannic acid or green tea was separately feeding to *Drosophila* larvae or adults (*w*^*1118*^, PPO1^△^, PPO2^△^, PPO1^△^/PPO2^△^) after being mixed with diet at different concentrations. The growth of larvae and adults were observed and counted every day.

### l-DOPA feeding for HPLC-MS analysis

To feed l-DOPA to *H. armigera* larvae, agar was melted and then brought to room temperature. Before the agar solidified, l-DOPA was added and mixed thoroughly (0.01 g/g agar). After solidification, the agar containing l-DOPA was fed to *H. armigera* larvae. Agar alone was also fed as a control. 5,6-dihydroxyindole (DHI; 5 mM, 50 μl) (Shanghai YuLan, China) and l-DOPA (2 mM, 50 μl) solutions were also injected into *H. armigera* larva separately. Silkworm larvae (V-2) were fed with approximately 10 μl l-DOPA solution (2 mM) or water. The above larvae were then bled into a tube containing some solid phenylthiourea (PTU). The collected hemolymph was centrifuged at 5,000 × g for 3 min at 4 °C to remove hemocytes. The plasma supernatant (80 μl) was mixed with 150 μl autoclaved water followed by high-speed vortexing and centrifugation at 10,000 g for 1 min at 4 °C. The supernatant was filtered using a 0.2 μm membrane filter. To take the foregut or midgut contents from silkworms or *H. armigera*, the above treated larvae were bled and dissected and fixed on a dissecting pan. The gut was washed using 0.85% NaCl several times to remove all hemolymph and then dried using tissue paper. Foregut or midgut contents from 3 larvae were mixed in a pre-weighed tube. Approximately 10 μg of fresh silkworm foregut contents or 50 μg midgut contents of *H. armigera* were suspended in 180 μl water and 20 μl saturated PTU solution. The mixture was vortexed at high-speed for 1 min and centrifuged at 10,000 × g for 3 min at 4 °C. The supernatant was filtered prior to analysis by mass spectrometry. When *H. armigera* larvae were injected with l-DOPA or DHI or water, plasma was collected at 5 and 30 min respectively, and the feces excreted at 30 min from 3 larvae were suspended in 200 μl water and vortexed at high speed for 1 min followed by centrifugation at 10,000 × g for 1 min at 4 °C. The supernatant was then filtered for analysis by mass spectrometry (see the supplemental file for detail).

## Additional Information

**How to cite this article**: Wu, K. *et al.* Plant phenolics are detoxified by prophenoloxidase in the insect gut. *Sci. Rep.* **5**, 16823; doi: 10.1038/srep16823 (2015).
